# Genetic Spectrum of *EYS*-associated Retinal Disease in a Large Japanese Cohort: Identification of Disease-associated Variants with Relatively High Allele Frequency

**DOI:** 10.1038/s41598-020-62119-3

**Published:** 2020-03-26

**Authors:** Lizhu Yang, Kaoru Fujinami, Shinji Ueno, Kazuki Kuniyoshi, Takaaki Hayashi, Mineo Kondo, Atsushi Mizota, Nobuhisa Naoi, Kei Shinoda, Shuhei Kameya, Yu Fujinami-Yokokawa, Xiao Liu, Gavin Arno, Nikolas Pontikos, Taro Kominami, Hiroko Terasaki, Hiroyuki Sakuramoto, Satoshi Katagiri, Kei Mizobuchi, Natsuko Nakamura, Go Mawatari, Toshihide Kurihara, Kazuo Tsubota, Yozo Miyake, Kazutoshi Yoshitake, Takeshi Iwata, Kazushige Tsunoda, JEGC study group, Toshihide Nishimura, Yoshihide Hayashizaki, Nobuhiro Shimozawa, Masayuki Horiguchi, Shuichi Yamamoto, Manami Kuze, Shigeki Machida, Yoshiaki Shimada, Makoto Nakamura, Takashi Fujikado, Yoshihiro Hotta, Masayo Takahashi, Kiyofumi Mochizuki, Akira Murakami, Hiroyuki Kondo, Susumu Ishida, Mitsuru Nakazawa, Tetsuhisa Hatase, Tatsuo Matsunaga, Akiko Maeda, Kosuke Noda, Atsuhiro Tanikawa, Syuji Yamamoto, Hiroyuki Yamamoto, Makoto Araie, Makoto Aihara, Toru Nakazawa, Tetsuju Sekiryu, Kenji Kashiwagi, Kenjiro Kosaki, Carninci Piero, Takeo Fukuchi, Atsushi Hayashi, Katsuhiro Hosono, Keisuke Mori, Kouji Tanaka, Koichi Furuya, Keiichirou Suzuki, Ryo Kohata, Yasuo Yanagi, Yuriko Minegishi, Daisuke Iejima, Akiko Suga, Brian P. Rossmiller, Yang Pan, Tomoko Oshima, Mao Nakayama, Megumi Yamamoto, Naoko Minematsu, Daisuke Mori, Yusuke Kijima, Kentaro Kurata, Norihiro Yamada, Masayoshi Itoh, Hideya Kawaji, Yasuhiro Murakawa

**Affiliations:** 1 Laboratory of Visual Physiology, Division of Vision Research, National Institute of Sensory Organs, National Hospital Organization Tokyo Medical Center, Tokyo, 152-8902 Japan; 2 Department of Ophthalmology, Keio University School of Medicine, Tokyo, 160-8582 Japan; 3 UCL Institute of Ophthalmology, London, EC1V 9EL UK; 4 Moorfields Eye Hospital, London, EC1V 2PD UK; 5 Department of Ophthalmology, Nagoya University Graduate School of Medicine, Nagoya, Aichi 466-8550 Japan; 6 Department of Ophthalmology, Kindai University Faculty of Medicine, Faculty of Medicine, Osaka-Sayama, Osaka 589-8511 Japan; 7 Department of Ophthalmology, The Jikei University School of Medicine, Tokyo, 105-8461 Japan; 8 Department of Ophthalmology, Mie University Graduate School of Medicine, Tsu, Mie 514-8507 Japan; 9 Department of Ophthalmology, Teikyo University, Tokyo, 173-8605 Japan; 10 Department of Ophthalmology, Miyazaki University, Miyazaki, Miyazaki 889-2192 Japan; 11 Department of Ophthalmology, Saitama Medical University, Moroyama, Saitama 350-0400 Japan; 12 Department of Ophthalmology, Nippon Medical School Chiba Hokusoh Hospital, Inzai, Chiba 270-1694 Japan; 13 Graduate School of Health Management, Keio University, Fujisawa, 252-0883 Japan; 14 Division of Public Health, Yokokawa Clinic, Suita, Osaka 564-0083 Japan; 15 Southwest Hospital/Southwest Eye Hospital, Third Military Medical University, Chongqing, 400030 China; 16 North East Thames Regional Genetics Service, UCL Great Ormond Street Institute of Child Health, Great Ormond Street NHS Foundation Trust, London, WC1N 1EH UK; 17 Department of Ophthalmology, The University of Tokyo, Tokyo, 113-8654 Japan; 18 Aichi Medical University, Nagakute, Aichi 480-1195 Japan; 19 Division of Molecular and Cellular Biology, National Institute of Sensory Organs, National Hospital Organization Tokyo Medical Center, Tokyo, 152-8902 Japan; 20 Department of Translational Medicine Informatics, St. Marianna University School of Medicine, Kawasaki, 216-8511 Japan; 21 RIKEN Preventive Medicine and Diagnosis Innovation Program, Wako, Saitama 351-0198 Japan; 22 National Institutes of Biomedical Innovation, Health and Nutrition, Tsukuba, 305-0843 Japan; 23 Department of Ophthalmology, Fujita Health University School of Medicine, Toyoake, 470-1192 Japan; 24 Department of Ophthalmology and Visual Science, Chiba University Graduate School of Medicine, Chiba, 263-8522 Japan; 25 Department of Ophthalmology, Matsusaka Central General Hospital, Matsusaka, 515-8566 Japan; 26 Saitama Medical Center, Dokkyo Medical University, Koshigaya, Saitama, 343-8555 Japan; 27 Fujita Health University Bantane Hospital, Nagoya, Aichi 454-8509 Japan; 28 Department of Ophthalmology, Kobe University Hospital, Kobe, Hyogo, 650-0017 Japan; 29 Osaka University Medical School, Suita, Osaka 565-0871 Japan; 30 Hamamatsu University School of Medicine, Hamamatsu, Shizuoka 431-3192 Japan; 31 Riken Center for Developmental Biology, Kobe, Hyogo, 650-0047 Japan; 32 Department of Ophthalmology, Gifu University Graduate School of Medicine, Gifu, Gifu 501-1112 Japan; 33 Department of Ophthalmology, Juntendo University Faculty of Medicine, Tokyo, 113-8431 Japan; 34 Department of Ophthalmology, University of Occupational and Environmental Health, Kitakyushu, Fukuoka 807-8556 Japan; 35 Laboratory of Ocular Cell Biology and Visual Science, Department of Ophthalmology, Faculty of Medicine and Graduate School of Medicine, Hokkaido University, Sapporo, Hokkaido 060-0808 Japan; 36 Hirosaki University Graduate School of Medicine, Hirosaki, Aomori 036-8562 Japan; 37 Graduate School of Medical and Dental Sciences, Niigata University, Niigata, Niigata 951-8510 Japan; 38 Division of Hearing and Balance Research, National Institute of Sensory Organs, National Hospital Organization, Tokyo Medical Center, Tokyo, 152-8902 Japan; 39 Hitoshi Ophthalmology Clinic, Nishinomiya, Hyogo 663-8184 Japan; 40 Kanto Central Hospital of the Mutual Aid Association of Public School Teachers, Tokyo, 158-8531 Japan; 41 Department of Ophthalmology, Graduate School of Medicine, Tohoku University, Sendai, Miyagi 980-8577 Japan; 42 Department of Ophthalmology, Fukushima Medical University School of Medicine, Fukushima, Fukushima 960-1247 Japan; 43 Department of Ophthalmology, University of Yamanashi, Chuo, Yamanashi 409-3898 Japan; 44 Center for Medical Genetics, Keio University School of Medicine, Tokyo, 160-8582 Japan; 45 Division of Genomic Technologies, Laboratory for Transcriptome Technology, RIKEN Center for Integrative Medical Sciences, Yokohama, Kanagawa 230-0045 Japan; 46 Division of Ophthalmology and Visual Science, Graduate School of Medical and Dental Sciences, Niigata University, Niigata, Niigata 950-2181 Japan; 47 Department of Ophthalmology, Graduate School of Medicine and Pharmaceutical Sciences, University of Toyama, Toyama, Toyama 930-0194 Japan; 48 Department of Ophthalmology, International University of Health and Welfare, Nasushiobara, Tochigi 329-2763 Japan; 49 Department of Ophthalmology, Nihon University Hospital, Tokyo, 101-8309 Japan; 50 Institute for Advanced Co-Creation Studies, Osaka University, Suita, Osaka 565-0871 Japan; 51 Department of Ophthalmology, Asahikawa Medical University, Asahikawa, Hokkaido 078-8802 Japan; 52 Graduate School of Agricultural and Life Sciences, The University of Tokyo, Tokyo, 113-8654 Japan; 53 RIKEN Preventive Medicine and Diagnosis Innovation Program, Yokohama, Kanagawa 230-0045 Japan

**Keywords:** Disease genetics, Genetic counselling

## Abstract

Biallelic variants in the *EYS* gene are a major cause of autosomal recessive inherited retinal disease (IRD), with a high prevalence in the Asian population. The purpose of this study was to identify pathogenic *EYS* variants, to determine the clinical/genetic spectrum of *EYS*-associated retinal disease (*EYS*-RD), and to discover disease-associated variants with relatively high allele frequency (1%-10%) in a nationwide Japanese cohort. Sixty-six affected subjects from 61 families with biallelic or multiple pathogenic/disease-associated *EYS* variants were ascertained by whole-exome sequencing. Three phenotype groups were identified in *EYS*-RD: retinitis pigmentosa (RP; 85.94%), cone-rod dystrophy (CORD; 10.94%), and Leber congenital amaurosis (LCA; 3.12%). Twenty-six pathogenic/disease-associated *EYS* variants were identified, including seven novel variants. The two most prevalent variants, p.(Gly843Glu) and p.(Thr2465Ser) were found in 26 and twelve families (42.6%, 19.7%), respectively, for which the allele frequency (AF) in the Japanese population was 2.2% and 3.0%, respectively. These results expand the phenotypic and genotypic spectrum of *EYS*-RD, accounting for a high proportion of *EYS*-RD both in autosomal recessive RP (23.4%) and autosomal recessive CORD (9.9%) in the Japanese population. The presence of *EYS* variants with relatively high AF highlights the importance of considering the pathogenicity of non-rare variants in relatively prevalent Mendelian disorders.

## Introduction

Inherited retinal disease (IRD) is one of the major causes of blindness in children and the working population in developed countries^1^. The prevalence of IRD is high among Mendelian disorders, with 1 in 3,500–4,000 individuals thought to be affected (Genetic Home References; https://ghr.nlm.nih.gov/condition)^1–3^.

In 2008, variants in the eyes shut homologue (*EYS*) gene (OMIM: 612424) were first identified as disease-causing for autosomal recessive retinitis pigmentosa (ARRP) by Abd El–Aziz *et al*. and Collins *et al*., independently^4,5^. Since the discovery, over 270 disease-associated variants have been reported according to the Human Gene Mutation Database (HGMD; 2018.4 version, https://portal.biobase-international.com)^6^.

*EYS* (NM_001142800.1) spans approximately 2 Mb of chr6q12 and encodes a protein with 3144 amino acids that contains 27 epidermal growth factor-like (EGF) domains and five Laminin G-like domains^4^. *EYS* is an orthologue of Drosophila’s spacemaker (spam), which has an essential role in the morphogenesis of photoreceptors^4,5,7^. Recent studies have shown that the absence of *EYS* causes disruptions of the photoreceptor structure and leads to cone-rod dystrophy (CORD) in zebrafish^8,9^. However, the exact molecular mechanism has not been clarified due to the limited resources of animal models, as the *EYS* gene is lacking in several rodent species^4,9,10^.

Several phenotypes have been associated with pathogenic *EYS* variants, such as RP and CORD; thus, “*EYS*-associated retinal disease (*EYS*-RD)” can be used as an accurate description for this disease, in consideration of the phenotypic spectrum^5,11–13^.

*EYS*-RD is currently thought to be one of the most prevalent IRDs in Asian and European populations^13–19^. Barragan *et al*. reported that *EYS*-RD accounted for 15.9% of ARRP in the Spanish population^17^, and Hosono *et al*. and Arai *et al*. reported a high prevalence (18%-23%) in Japanese cohorts of RP patients^15,20^. However, the pathogenicity of the *EYS* variants and the accurate prevalence of *EYS*-RD, as well as what underlies this high prevalence of *EYS*-RD, are still uncertain due to the lack of large cohort studies.

Recently, disease-associated variants with relatively high allele frequency (AF; >1% in the general population) have been identified in *ABCA4*-associated retinal disease, representing one of the most prevalent IRDs^21–24^. A survey of variants with relatively high AF has become increasingly important to obtain an accurate genetic diagnosis in the era of next-generation sequencing. This is because high-throughput genetic screening technologies with automated variant filtering remove variants with a population frequency above the selected cut-off^23,24^. The analysis of variants with relatively high AF is anticipated to be more relevant for relatively prevalent Mendelian disorders such as IRD, especially in an isolated population such as the Japanese population, given the potential presence of founder variants.

The purpose of this study was to determine pathogenic/disease-associated *EYS* variants utilizing an analysis of variants with relatively high AF and to clarify the clinical and genetic spectrum of *EYS*-RD in a large nationwide Japanese cohort. The AF of *EYS* variants in affected subjects and in the general population were also investigated to estimate the AF-based prevalence of *EYS*-RD.

## Results

### Participants, demographics and visual acuity

In total, 66 affected subjects from 61 families with *EYS*-RD were included in this study. Co-segregation analysis was performed within 20 families. Thirty-six family members from 61 families underwent clinical examination and whole-exome sequencing. The pedigree charts showing the clinical and genetic status of each affected and unaffected subject are presented in Supplementary Fig. S1. The phenotypic and genotypic features of the affected subjects are summarized in Supplementary Table S1.

**Figure 1:**
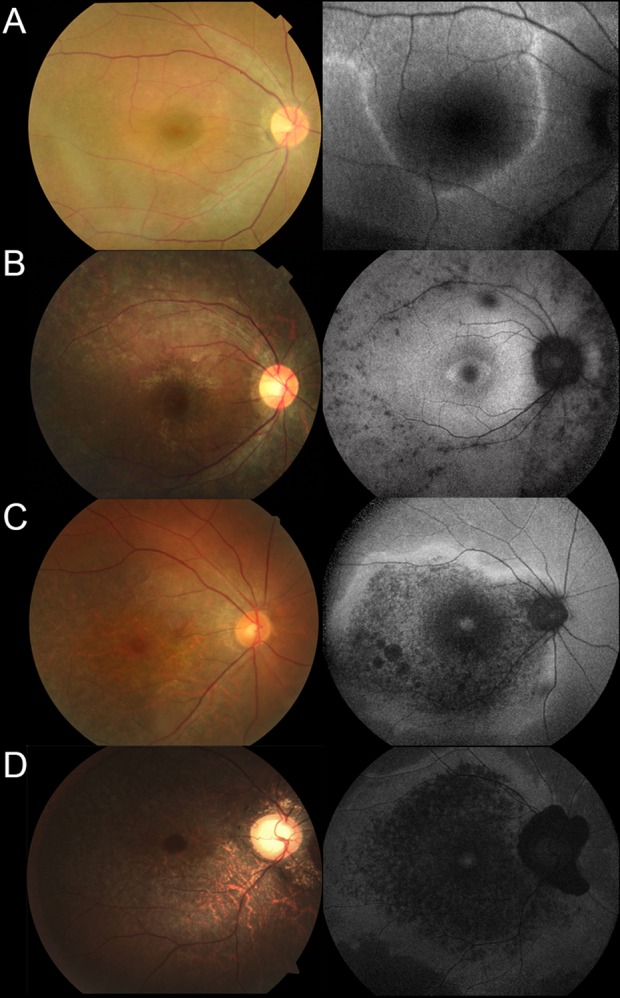
Fundus and autofluorescence findings of four representative cases with *EYS*-associated retinal disease (*EYS*-RD). (**A**) A 43-year-old female (2-III:2) diagnosed with retinitis pigmentosa (RP) harbouring homozygous variants (c.[2528 G > A];[2528 G > A], p.[(Gly843Glu)];[(Gly843Glu)]) in the *EYS* gene, showing retinal atrophic changes along the vessel arcade. (**B**) A 17-year-old male (22-II:2) diagnosed with RP harbouring homozygous variants (c.[4957dupA];[4957dupA], p.[(Ser1653Lysfs*2)];[(Ser1653Lysfs*2)]) in the *EYS* gene, showing retinal atrophic changes outside the vessel arcade. (**C**) A 50-year-old female (11-II:1) diagnosed with cone-rod dystrophy (CORD) harbouring two pairs of homozygous variants (c.[2528 G > A;c.7394 C > G];[2528 G > A;c.7394 C > G], p.[(Gly843Glu);(Thr2465Ser)];[(Gly843Glu);(Thr2465Ser)]) with relatively high allele frequency (AF) in the *EYS* gene, showing retinal atrophic changes within the vessel arcade. (**D**) A 39-year-old male (23-II:1) diagnosed with CORD harbouring compound heterozygous variants (c.[4957dupA];[8805 C > A], p.[(Ser1653Lysfs*2)];[(Tyr2935*)]) in the *EYS* gene, showing retinal atrophic changes within the vessel arcade.

The detailed clinical data are presented in Supplementary Table S2. All 66 affected subjects were Japanese, and there were 36 females and 30 males. The median age of 66 affected subjects was 46.0 years at examinations (range, 11.0–84.0), with a median age of onset of 21.0 (range, 1.0–65.0). The median best-corrected visual acuity (BCVA) was 0.1 logarithm of minimum angle of resolution (LogMAR) unit (range, −0.18–1.8). There were three eyes with hand motion, two with light perception, and one with non-light perception. Retinal images of four representative cases caused by prevalent variants in the *EYS* gene are presented in Fig. 1.

### Phenotype subgroups

The described clinical diagnosis was RP in 52 families (57 affected subjects), CORD in seven families (seven affected subjects), and early-onset retinitis pigmentosa or Leber congenital amaurosis (EORP/LCA) in two families (two affected subjects). The median age of onset and the median age at examination of 57 affected subjects with RP were 20.0 (range 1.0–65.0) and 46.5 (range 17.0–84.0), respectively. The median age of onset and the median age at examination of seven affected subjects with CORD were 40.0 (range 29.0–43.0) and 46.0 (range 39.0–76.0), respectively. In three families, different clinical diagnoses were described for the affected subjects within the family (Families 2, 3 and 28). Candidate variants were identified in the *CACNA1F* gene (OMIM; 300110), the *TOPORS* gene (OMIM; 609507), the *RIMS1* gene (OMIM; 606629), the *DRAM2* gene (OMIM; 613360), and the *RP1L1* gene (OMIM; 608581) gene, respectively. (Supplementary Table S1, Supplementary Figs. S1 and S2).

### *EYS* variants and *in silico* molecular genetic analysis

Whole-exome sequencing was performed for all the genomic DNA samples of 102 subjects from 61 families. The mean depth (±standard deviation) was 81.87 ± 25.15× and the mean coverage for the targeted regions was 96.03 ± 3.02% with a depth higher than 15×. The mean depth/coverage for the detected *EYS* variants in this study is listed in Table 1. No copy number variants associated with the disease were identified.

**Table 1:** Summary of genetic analyses for 26 *EYS* variants.

No.	Variant	Variant type	Family count (%*)	AC^*^	AF^*^	Mean read depth	Coverage (≥15 reads)	AF in general databases				ACMG classification	Reference
								HGVD	iJGVD 3.5k	gnomAD EA	gnomAD Total		
1	c.2528 G > A, p.(Gly843Glu)	Missense	26 (42.6%)	32	26.23%	37.24	99.87%	2.25%	1.7000%	0.0391%	0.0026%	LP	^25^
2	c.4957dupA, p.(Ser1653Lysfs*2)	Frameshift	22 (36.1%)	29	23.77%	38.27	100.00%	0.2084%	0.0000%	0.0098%	0.0007%	P	^15,20,25,32^
3	c.8805 C > A, p.(Tyr2935*)	Nonsense	14 (23.0%)	17	13.93%	37.11	100.00%	0.2901%	0.1700%	0.0293%	0.0020%	P	^15,20,25^
4	c.7394 C > G, p.(Thr2465Ser)	Missense	12 (19.7%)	15	12.30%	36.70	99.74%	3.0468%	2.9000%	0.1465%	0.0126%	US	Novel^†^^20^
5	c.6557 G > A, p.(Gly2186Glu)	Missense	7 (11.5%)	7	5.74%	35.09	99.15%	0.0000%	0.0000%	0.0497%	0.0035%	LP	^14,20,32^
6	c.6563 T > C, p.(Ile2188Thr)	Missense	5 (8.2%)	6	4.92%	32.15	97.12%	0.0000%	0.1000%	NA	NA	US	^15^
7	c.1211dupA, p.(Asn404Lysfs*3)	Frameshift	3 (4.9%)	3	2.46%	48.45	99.80%	0.0000%	0.0000%	0.0000%	0.0016%	P	^25,33^
8	c.632 G > A, p.(Cys211Tyr)	Missense	2 (3.3%)	2	1.64%	35.97	99.41%	0.0000%	0.0300%	NA	NA	LP	^15^
9	c.7665_7666del, p.(Tyr2555*)	Nonsense	2 (3.3%)	2	1.64%	33.26	93.72%	0.0000%	0.0000%	NA	NA	P	^25^
10	c.5644 + 5 G > A	Splicing	1 (1.6%)	1	0.82%	31.63	93.85%	0.0000%	0.0000%	NA	NA	P	^34^
11	c.3809 T > G, p.(Val1270Gly)	Missense	1 (1.6%)	1	0.82%	35.88	99.93%	0.4570%	0.0455%	0.0492%	0.0033%	US	^25^
12	c.5027 C > G, p.(Ser1676*)	Nonsense	1 (1.6%)	1	0.82%	35.37	98.50%	0.0000%	0.0000%	0.0000%	0.0000%	P	This study
13	c.7002 C > A, p.(Cys2334*)	Nonsense	1 (1.6%)	1	0.82%	27.08	90.26%	0.0000%	0.0100%	NA	NA	P	^35^
14	c.6714del, p.(Ile2239Serfs*17)	Frameshift	1 (1.6%)	1	0.82%	27.97	91.69%	0.0000%	0.0000%	0.0098%	0.0039%	P	^5,17,27,35^
15	c.1485_1493delinsCGAAAAG, p.(Val495Glufs*13)	Frameshift	1 (1.6%)	1	0.82%	33.67	99.22%	0.0000%	0.0000%	NA	NA	P	^25^
16	c.137 C > T, p.(Thr46Ile)	Missense	1 (1.6%)	1	0.82%	38.23	99.28%	0.0000%	0.0000%	NA	NA	US	This study
17	c.9186_9187del, p.(Asn3062Lysfs*9)	Frameshift	1 (1.6%)	1	0.82%	39.32	99.61%	0.0000%	0.0000%	0.0000%	0.0013%	P	^36^
18	c.8608 A > T, p.(Asn2870Tyr)	Missense	1 (1.6%)	1	0.82%	48.93	100.00%	0.0000%	0.0000%	NA	NA	US	This study
19	c.141 A > T, p.(Glu47Asp)	Missense	1 (1.6%)	1	0.82%	38.97	100.00%	0.0000%	0.0100%	NA	NA	US	^15,35^
20	c.4022del, p.(Ser1341Phefs*11)	Frameshift	1 (1.6%)	1	0.82%	43.99	100.00%	0.0000%	0.0000%	NA	NA	P	^35^
21	c.8278 C > T, p.(Arg2760Cys)	Missense	1 (1.6%)	1	0.82%	39.08	99.80%	0.0000%	0.0000%	0.0000%	0.0000%	US	This study
22	c.8516dupA, p.(Asn2839Lysfs*2)	Frameshift	1 (1.6%)	1	0.82%	24.03	87.22%	0.0000%	0.0000%	NA	NA	P	This study
23	c.7609 G > A, p.(Ala2537Thr)	Missense	1 (1.6%)	1	0.82%	33.56	98.63%	0.5013%	0.2300%	0.2678%	0.0266%	US	^36,37^
24	c.2000G > A, p.(Arg667His)	Missense	1 (1.6%)	1	0.82%	35.18	99.87%	0.0000%	0.0327%	0.0000%	0.0295%	US	^38^
25	c.7919 G > A, p.(Trp2640*)	Nonsense	1 (1.6%)	1	0.82%	41.19	100.00%	0.0000%	0.0600%	0.0000%	0.0027%	P	^4,17,39^
26	c.7392dupT, p.(Thr2465Tyrfs*12)	Frameshift	1 (1.6%)	1	0.82%	41.27	100.00%	0.0000%	0.0000%	NA	NA	P	This study

AC = allele count; AF = allele frequency; EA = East Asian; ACMG = American College of Medical Genetics and Genomics; P = pathogenic; LP = likely pathogenic; US = uncertain significance; HGVD = Human Genetic Variation Database (http://www.genome.med.kyoto-u.ac.jp/SnpDB/; accessed on July 1, 2017); iJGVD 3.5k=Integrative Japanese Genome Variation 3.5k (https://ijgvd.megabank.tohoku.ac.jp/download_3.5kjpn/; accessed on July 1, 2017); gnomAD=the Genome Aggregation Database (http://gnomad.broadinstitute.org/; accessed on 1st of August, 2018).

^*^Number is this cohort; ^†^: a variant listed in the cited reference, but not associated with the disease.

**Figure 2:**
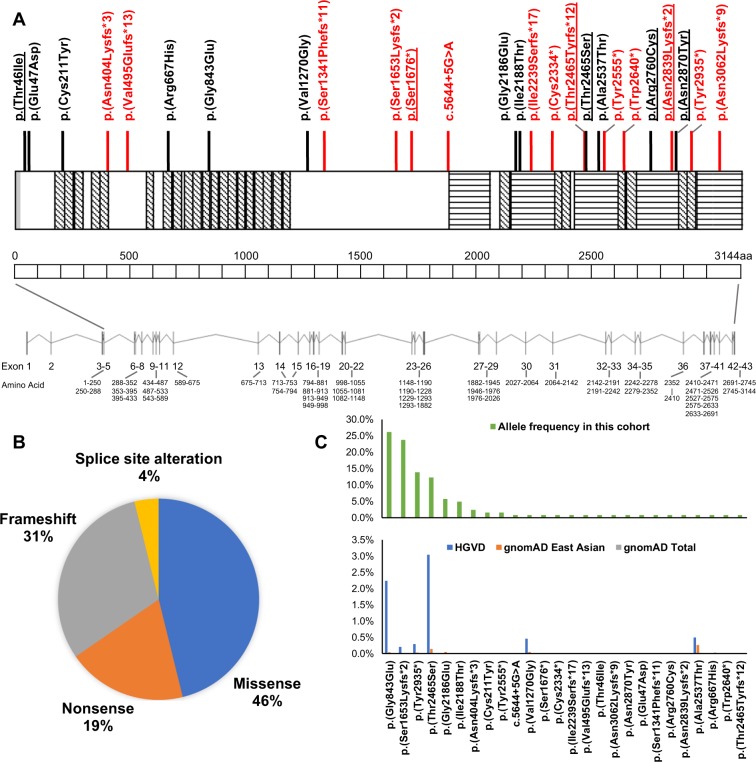
*EYS* variants detected in a Japanese cohort with inherited retinal disease (IRD). (**A**) A schematic genetic and protein structure of *EYS* and the location of the detected variants in this study. The *EYS* gene (ENST00000503581.5) contains 43 exons. Exons 4 to 43 encode a 3144-amino acid protein containing 27 epidermal growth factor-like domains (highlighted with diagonal lines) and five laminin G-like domains (highlighted with horizontal lines) as well as one N-terminal signal peptide (highlighted with grey). Truncating variants (nonsense, frameshift, and splice site alteration) are shown in red, and missense variants are shown in black. Novel variants identified in this study are underlined. (**B**) Distribution of the types of the detected variants. In total, 26 variants were identified, including twelve missense variants (46%), eight frameshift variants (31%), five nonsense variants (19%) and one splicing site alteration variant (4%). (**C**) Allele frequency (AF) of the detected variants in this *EYS*-RD cohort and in the general population presented in public databases. Top: The AF in this *EYS* affected cohort. c.2528 G > A (p.(Gly843Glu)), c.4957dupA (p.(Ser1653Lysfs*2)), c.8805 C > A (p.(Tyr2935*)) and c.7394 C > G (p.(Thr2465Ser)) are the four most prevalent variants, with AFs of 26.23%, 23.77%, 13.93%, and 12.30%, respectively. Bottom: The AF in the general population in the two public databases: the Genome Aggregation Database (gnomAD; a database for the ethnic and the total general population) and the Human Genetic Variation Database (HGVD; a database for the Japanese general population). The AF of the four most prevalent variants provided by the HGVD/gnomAD East Asian/gnomAD total databases was 2.25%/0.04%/0.00%, 0.21%/0.01%/0.00%, 0.29%/0.03%/0.00%, and 3.05%/0.15%/0.01%, respectively, for c.2528 G > A (p.(Gly843Glu)), c.4957dupA (p.(Ser1653Lysfs*2)), c.8805 C > A (p.(Tyr2935*)) and c.7394 C > G (p.(Thr2465Ser)).

Twenty-six *EYS* variants were identified in total. Six variants have never been reported, and one variant (c.7394 C > G, p.(Thr2465Ser)) has never been associated with the specific phenotype of RP/CORD/LCA. The detected variants are widely distributed in the *EYS* gene (Fig. 2). There were twelve missense variants, eight frameshift variants, five nonsense variants, and one variant with splice site alteration. The genetic results are summarized in Table 1 and Supplementary Table S1.

Detailed results of the *in silico* analyses of 26 variants are presented in Supplementary Table S3. Thirteen variants were classified as pathogenic, four were likely pathogenic, and nine were variants of uncertain significance (VUS). Eight VUS were found with likely pathogenic/pathogenic variants or variants previously reported as likely pathogenic elsewhere. One variant (c.8608 A > T, p.(Asn2870Tyr)) was found with the recurrent relatively high AF variant (c.7394 C > G, p.(Thr2465Ser)).

The AF of all 26 variants detected in this affected cohort and in the general population is presented in Table 1 and Fig. 2. The AF of *EYS* variants ranged from 0.00% to 3.05% in the Human Genetic Variation Database (HGVD), 0.00% to 0.27% in the East Asian in the Genome Aggregation Database (gnomAD), and 0.00% to 0.03% in total in the gnomAD database. The four most prevalent variants were c.2528 G > A (p.(Gly843Glu)), c.4957dupA (p.(Ser1653Lysfs*2)), c.8805 C > A (p.(Tyr2935*)), and c.7394 C > G (p.(Thr2465Ser)), with an AF in this affected *EYS* cohort of 26.23% (32/122), 23.77% (29/122), 13.93% (17/122), and 12.30% (15/122), respectively. The AF of these four most prevalent variants in the HGVD/gnomAD East Asian/gnomAD total was 2.25%/0.04%/0.00%, 0.21%/0.01%/0.00%, 0.29%/0.03%/0.00%, and 3.05%/0.15%/0.01%, respectively. A considerably higher AF in the Japanese population was observed in these four variants than in the other populations. There were two more variants with higher AF ( > 0.45%) in the general Japanese population: c.3809 T > G (p.(Val1270Gly)) and c.7609 G > A (p.(Ala2537Thr)), with the AF provided by the HGVD/gnomAD East Asian/gnomAD total being 0.46%/0.05%/0.00% and 0.50%/0.27%/0.03%, respectively.

## Discussion

The genetic spectrum of *EYS*-RD is illustrated in a well-characterized large Japanese cohort of 61 families. The identification of variants with relatively high AF confirmed by the co-segregation analysis in multiple families helped to clarify the high proportion of *EYS*-RD in the IRDs of the Japanese population; 23.4% of AR or sporadic RP (52/222) and 9.9% of AR or sporadic CORD (7/71).

### Two *EYS* variants with allele frequencies higher than 1%

Two variants with relatively high AF (>1% in the general population) were confirmed in our cohort: p.(Gly843Glu) and p.(Thr2465Ser). All the subjects harbouring these variants in a homozygous or compound heterozygous status in the Japan Eye Genetics Consortium (JEGC; http://www.jegc.org/) study cohort of 1302 subjects from 729 families demonstrated retinal dystrophy, which supports the disease causation/association of these two variants.

The variant p.(Gly843Glu) was first described by Iwanami *et al*. in 2012. Five subjects with this variant found with the other proven truncating variants, such as p.(Ser1653Lysfs*2) and p.(Ser2428*), were presented in this report^25^. In our study, there were five families homozygous for this variant, showing various phenotypic findings in the spectrum of RP. In the HGVD database, there is one subject homozygous for this variant out of 1207 subjects (1/1207, 0.08%) with no registered diseases on the records for whom no further ophthalmic information is available^26^. Given the variable disease onset and phenotype associated with this variant, it is still uncertain whether this subject will develop visual defects in the future. The AF of this variant in our molecularly proven ARRP cohort of 112 families (32/224; 14.3%) was significantly higher than that in the general Japanese population (53/2361, 2.25%; HGVD) calculated with Fisher’s exact test (P < 0.001), as implied by the previous studies in the Japanese population^19,25^. Moreover, the AF in the general Japanese population was approximately 50/1000 times higher than that in the East Asian/total population of gnomAD. The pathogenicity of this variant is not fully proven; however, a founder effect in the Japanese population should be considered to explain this most prevalent disease-associated allele.

The other variant with relatively high AF (p.(Thr2465Ser)) was first described by Hosono *et al*. in 2012 as a possible non-pathogenic variant with the allele frequency of affected (8/200; 4.0%) and normal subjects (2/192; 1.0%)^20^. In our cohort, twelve families harboured this variant and no other candidate variants in any other known retinal disease-associated gene. Three of these twelve families had proven biallelic *EYS* variants confirmed by the co-segregation analysis. It is of note that five alleles of this variant were associated with CORD. In addition, this variant was found *in cis* with the p.(Gly843Glu) variant in two families with an additional family harbouring three candidate unsegregated variants. The AF of this variant in our molecularly confirmed ARRP cohort (15/224; 6.70%) was higher than that of the general Japanese population (67/2203, 3.04%; HGVD) calculated with Fisher’s exact test, which reached a statistically significant value (P = 0.01). The AF in the general Japanese population was approximately 200/2000 times higher than those in the East Asian/total population, respectively. In the HGVD database, there are two subjects (2/1207, 0.17%) homozygous for this variant with no available ophthalmic information. The results of *in silico* analysis and comparison analysis between the AF of the affected cohort and the general population suggest some supporting evidence for the disease causation, and a founder effect in the Japanese population could also be considered for this prevalent allele that is possibly associated with IRDs.

### The *EYS* gene and the high prevalence of IRDs in the Japanese population

Two prevalent truncating variants (p.(Ser1653Lysfs*2) and p.(Tyr2935*)) were also frequently found in our cohort. As previously described, these two variants have a higher AF in the Japanese population than in other populations^15,19,20,25^. Together with the other two variants with high AF (p.(Val1270Gly) and p.(Ala2537Thr); AF > 0.45%) in the Japanese general population, several frequent variants especially prevalent in the Japanese population were determined in this study.

The total value of AF of the total detected *EYS* variants was 6.75% in the general Japanese population. Given this number, the estimated prevalence of subjects at risk for *EYS*-RD in Japan should be higher than the current estimated value of 1 in 3500–4000 for RP. However, it is of note that the genetic risk does not perfectly correspond to the prevalence of the disease in the real world, as shown for the most prevalent *ABCA4* variant (p.(Asn1868Ile) in the European population (AF > 6.7%)^21^.

### Genotype-phenotype association of *EYS*-RD

Three phenotype groups were identified in our cohort: RP (85.9%), CORD (10.9%), and EORP/LCA (3.1%). There were only a few patients with *EYS*-CORD reported to date in the previous literature; however, our *EYS*-RD cohort provided the largest number of CORD patients associated with *EYS* variants^5,11–13^. Seven out of 45 molecularly proven cases of ARCORD in the JEGC cohort are caused by biallelic or putative biallelic *EYS* variants (7/45, 15.6%). This fact highlighted that *EYS* should be the major IRD gene in the Japanese population, with the a significantly higher prevalence than that in the European population^5,16,17,27^.

There was no clear genotype-phenotype association/correlation between RP/CORD/EORD due to the limited number of CORD and EORD/LCA cases. Although further detailed analysis is needed for accurate assessment, both of the two aforementioned variants with relatively high AF are associated with either RP or CORD; thus, the prediction of predominant functional failure (rod or cone) seems hard based on the genotype. It is noteworthy that even patients with the identical genotype presented with the contrasting clinical phenotypes (RP/CORD), which suggests the possible presence of modifiers outside of the *EYS* gene that contribute to the disease presentation.

There were seven families with multiple disorders (Supplementary Fig. S2) or non-AR inheritance in our cohort. Conclusive genetic diagnosis is still unavailable in four affected subjects with limited clinical information from three families (Families 5, 8 and 16). Whole-exome sequencing was not performed in three subjects from two families (Families 5 and 16). Given the presence of variants with a relatively high AF, such coincidence with the other *EYS* variants or other pathogenic variants in the non-*EYS* genes should be considered in the clinical/genetic diagnosis of IRD. For this reason, comprehensive gene screening is helpful to elucidate the cause of complicated phenotypes in such families.

### Limitations of this study

There are limitations to this study. First, the molecular mechanisms of disease causation for most variants have not yet been known, and the clinical effect of the variants (e.g., functional loss by a single variant, acting as a modifier, complexing with missing disease-causing variants, and others) is poorly understood. Further functional analysis is needed to conclude the disease causation of each variant. Second, the identification of copy number variants is technically hard with the results of whole-exome sequencing; thus, the possible presence of copy number variants was not completely excluded in our study. As previously reported^28,29^, it is crucial to examine the structural variants in the *EYS* gene. Third, the AF data of general populations were not studied in the detail due to the limited data resources of ophthalmic findings and natural history, which should be valuable to assess the clinical effect of each variant in subjects at risk in the real world, especially in relatively prevalent IRDs with diverse onset and phenotype. Last, the identification of background ethnicity for each variant was not available in this study. Extensive genomic analysis with detailed haplotype information could delineate the ethnic specificity of *EYS*-RD.

In conclusion, the phenotypic and genotypic characteristics of *EYS*-RD were determined in this largest cohort of the Japanese population. The presence of variants with a relatively high AF in a specific population requires the survey of non-rare variants in consideration of founder effects, especially in relatively prevalent Mendelian disorders.

## Methods

The protocol of this study adhered to the tenets of the Declaration of Helsinki and was approved by the ethics committee of the participating institutions from Japan; National Institute of Sensory Organs, National Hospital Organization Tokyo Medical Center (Reference; R18–029). A signed informed consent was obtained from all subjects.

### Participants and clinical investigation

Patients with a clinical diagnosis of IRD and available genetic data were studied between 2008 and 2018 as part of the JEGC (http://www.jegc.org/)^30^. A total of 1302 subjects from 729 families, including 222 families with autosomal recessive or sporadic RP and 71 families with autosomal recessive or sporadic CORD, registered in the JEGC cohort were surveyed. Clinical information is available via the NISO online database, including medical history, family history, ethnicity, chief complaints of visual symptoms, the onset of disease, the best-corrected decimal visual acuity converted to the LogMAR unit, fundoscopy, fundus photography, autofluorescence imaging, and phenotypic categorization.

### *EYS* variant detection

Genomic DNA was extracted from the peripheral blood of all affected subjects and the available unaffected family members with the Gentra Puregene Blood Kit (Qiagen, Tokyo, Japan). Whole-exome sequencing with targeted analysis of retinal disease-associated genes (RetNet; https://sph.uth.edu/retnet/home.htm; accessed on 1 January 2017) was performed on the affected subjects and unaffected family members according to previously published methods^30^. Briefly, paired-end sequence library construction and exome capturing were performed with the Agilent Bravo automated liquid-handling platform with SureSelect XT Human All Exon V3–5+UTRs kit (Agilent Technologies, Santa Clara, CA, USA). Enriched libraries were sequenced with the Illumina HiSeq. 2000/2500 sequencer (San Diego, CA, USA; read length 2×101 bp). Reads were aligned to the University of California, Santa Cruz (USCS; California, United States) human genome 19 reference sequence with Burrows-Wheeler Aligner software. Duplicated reads were removed by the Picard MarkDuplicates module, and mapped reads around insertion-deletion polymorphisms (INDELs) were realigned by the Genome Analysis Toolkit (GATK) Version 3.0. Base-quality scoring was recalibrated by the GATK. Mutation calling was performed by the GATK Unified Genotyper module. Called single-nucleotide variants (SNVs) and INDELs were annotated by the snpEff software (snpEff score; “high”, “moderate” or “low”). The XHMM (eXome-Hidden Markov Model; https://atgu.mgh.harvard.edu/xhmm/) tool was applied for the detection of copy number variations. The read depth and coverage of the targeted regions were also confirmed with Integrate Genome Viewer (IGV; http://software.broadinstitute.org/software/igv/). All called SNVs and INDELs of the RetNet genes were selected for further analysis. Variants with the read depths higher than 15× were selected for this study.

The identified variants with an allele frequency of less than 1% in the HGVD (http://www.genome.med.kyoto-u.ac.jp/SnpDB/; accessed on 1 July 2017), and Integrative Japanese Genome Variation (iJGVD 3.5k, https://ijgvd.megabank.tohoku.ac.jp/download_3.5kjpn/; accessed on 1 July 2017), which are two allele frequency databases specific for the Japanese population, were filtered. Only for the two autosomal recessive genes with high prevalence (*EYS* and *ABCA4*), the identified variants were filtered with an AF of less than 10.0% in the HGVD to avoid missing the pathogenic/disease-associated variants with a relatively high AF (1%-10%). Together with phenotypic features and inheritance, as well as co-segregation, disease-causing/disease-associated variants were determined from the called variants in the retinal disease-associated genes.

### *In silico* molecular genetic analysis

Sequence variant nomenclature was performed according to the guidelines of the Human Genome Variation Society (HGVS; version 2.0; http://varnomen.hgvs.org/). The AF of all detected variants in the HGVD, iJGVD, 1000 Genome Project (http://www.internationalgenome.org/; accessed on 1 August 2018), and gnomAD (http://gnomad.broadinstitute.org/; accessed on 1 August 2018) databases was established. All variants were analysed using two general prediction programmes and three functional prediction programmes: MutationTaster (http://www.mutationtaster.org/; accessed on 1 August 2018), FATHMM (http://fathmm.biocompute.org.uk/9; accessed on 1 August 2018), SIFT (https://www.sift.co.uk/; accessed on 1 August 2018), PROVEAN (http://provean.jcvi.org/index.php; accessed on 1 August 2018), and PolyPhen 2 (http://genetics.bwh.harvard.edu/pph2/; accessed on 1 August 2018). Evolutional conservation scores were calculated from the UCSC database (https://genome.ucsc.edu/index.html; accessed on 1 August 2018). Variant classification was performed according to the guidelines of the American College of Medical Genetics and Genomics (ACMG) for all detected variants^31^.

## Supplementary information

Supplementary Figure S1 and Supplementary Figure S2.

Supplementary Table S1.

Supplementary Table S2.

Supplementary Table S3.
