# Supplementary material for: Genetic Spectrum of EYS-associated Retinal Disease in a Large Japanese Cohort: Identification of Disease-associated Variants with Relatively High Allele Frequency
Source: Sci Rep. 2020 Mar 26;10:5497. doi: 10.1038/s41598-020-62119-3 (PMC7099090; doi:10.1038/s41598-020-62119-3)
Supplement: Supplementary file 1 — Supplementary Figure S1 and Supplementary Figure S2. [file 41598_2020_62119_MOESM1_ESM.pdf]

SUPPLEMENTARY INFORMATION

Genetic Spectrum of *EYS*-associated Retinal Disease in a Large Japanese Cohort: Identification of Disease-associated Variants with Relatively High Allele Frequency.

Lizhu Yang[1,2*], Kaoru Fujinami[1-4*], Shinji Ueno[5], Kazuki Kuniyoshi[6], Takaaki Hayashi[7], Mineo Kondo[8], Atsushi Mizota[9], Nobuhisa Naoi[10], Kei Shinoda[9,11], Shuhei Kameya[12], Yu Fujinami-Yokokawa[1,13,14], Xiao Liu[1,2,15], Gavin Arno[1, 3, 4, 16], Nikolas Pontikos[1,3,4], Taro Kominami[5], Hiroko Terasaki[5], Hiroyuki Sakuramoto[6], Satoshi Katagiri[7], Kei Mizobuchi[7], Natsuko Nakamura[1,9,17], Go Mawatari[10], Toshihide Kurihara[2], Kazuo Tsubota[2], Yozo Miyake[18], Kazutoshi Yoshitake[19], Takeshi Iwata[19], Kazushige Tsunoda[1]; on behalf of JEGC study group.

*Equal contributions and regarded as joint first authors

[1]Laboratory of Visual Physiology, Division of Vision Research, National Institute of Sensory Organs, National Hospital Organization Tokyo Medical Center, Tokyo, 152-8902, Japan.

[2]Department of Ophthalmology, Keio University School of Medicine, Tokyo, 160-8582, Japan.

[3]UCL Institute of Ophthalmology, London, EC1V 9EL, UK.

[4]Moorfields Eye Hospital, London, EC1V 2PD, UK.

[5]Department of Ophthalmology, Nagoya University Graduate School of Medicine, Nagoya, Aichi, 466-8550, Japan

[6]Department of Ophthalmology, Kindai University Faculty of Medicine, Faculty of Medicine, Osaka-Sayama, Osaka, 589-8511, Japan

[7]Department of Ophthalmology, The Jikei University School of Medicine, Tokyo, 105-8461, Japan

[8]Department of Ophthalmology, Mie University Graduate School of Medicine, Tsu, Mie, 514-8507, Japan

[9]Department of Ophthalmology, Teikyo University, Tokyo, 173-8605, Japan

[10]Department of Ophthalmology, Miyazaki University, Miyazaki, Miyazaki, 889-2192, Japan

[11]Department of Ophthalmology, Saitama Medical University, Moroyama, Saitama, 350-0400, Japan

[12]Department of Ophthalmology, Nippon Medical School Chiba Hokusoh Hospital, Inzai, Chiba, 270-1694, Japan.

[13]Graduate School of Health Management, Keio University, Fujisawa, 252-0883, Japan.

[14]Division of Public Health, Yokokawa Clinic, Suita, Osaka, 564-0083, Japan

[15]Southwest Hospital/Southwest Eye Hospital, Third Military Medical University, Chongqing, 400030, China

[16]North East Thames Regional Genetics Service, UCL Great Ormond Street Institute of Child Health, Great Ormond Street NHS Foundation Trust, London, WC1N 1EH, UK.

[17]Department of Ophthalmology, The University of Tokyo, Tokyo, 113-8654, Japan.

[18]Aichi Medical University, Nagakute, 480-1195, Aichi, Japan

[19]Division of Molecular and Cellular Biology, National Institute of Sensory Organs, National Hospital Organization Tokyo Medical Center, Tokyo, 152-8902, Japan.

Correspondence:

Dr. Kaoru Fujinami, Laboratory of Visual Physiology, Division of Vision Research, National Institute of Sensory Organs, National Hospital Organization Tokyo Medical Center, 2-5-1 Higashigaoka, Meguro-ku, Tokyo 152-8902, Japan; k.fujinami@ucl.ac.uk; TEL: +81 (0)3-3411-0111; FAX: +81 (0)3-3412-9811.

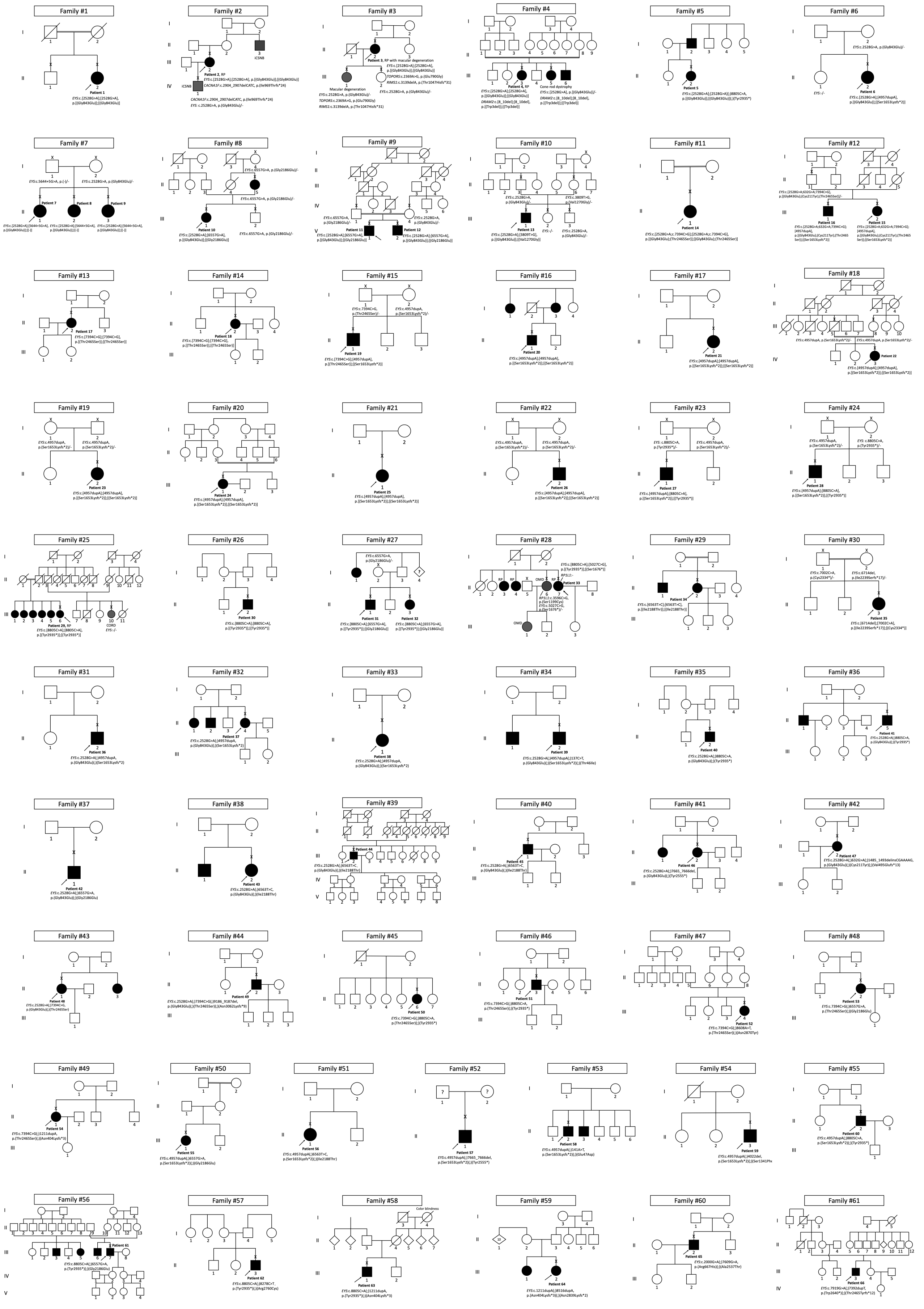

Supplementary Figure S1. Pedigrees of the 61 *EYS*-RD families.

The solid squares (male) and circles (female) represent the affected patients, and unaffected family members are represented by white icons. The rhombus represents subjects with unknown gender. The question mark indicates subjects whose affected/unaffected status is uncertain. The slash symbol indicates deceased individuals. The generation number is shown on the left. The proband of each pedigree is marked by an arrow, and the clinically examined individuals are indicated by a cross.

iCSNB=incomplete congenital night blindness; RP=retinitis pigmentosa; OMD=occult macular dystrophy.

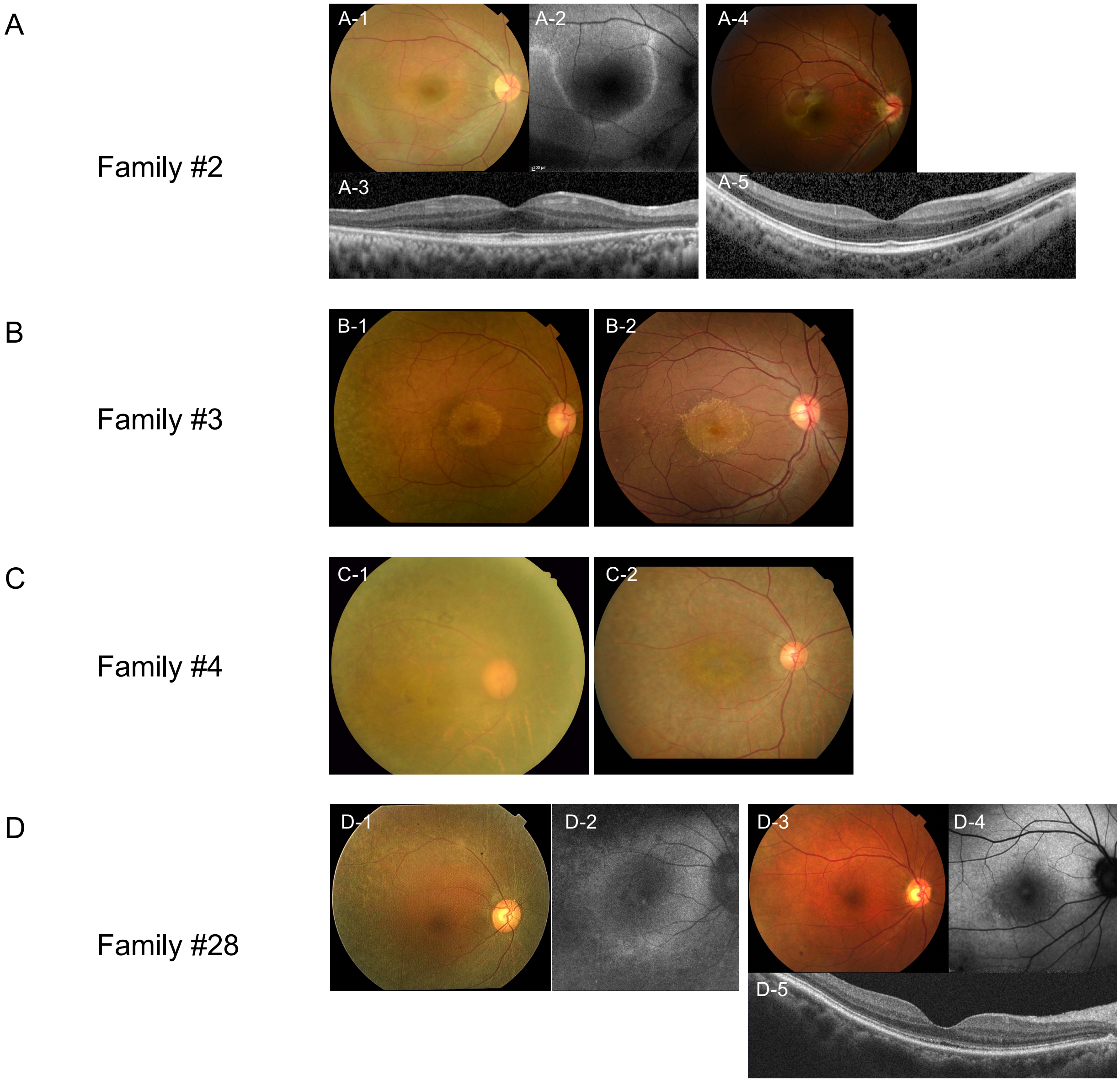

Supplementary Figure S2. Four families with different clinical diagnoses and disease-causing genes.
A (Family #2): The proband (2-III:2) was diagnosed with retinitis pigmentosa (RP) (A-1: fundus photograph; A-2: fundus autofluorescence (FAF); and A-3: optical coherence tomography (OCT)). One homozygous disease-causing *EYS* variant was identified. The affected son (2-IV:1) of this proband shows the phenotype of incomplete congenital stationary night blindness (iCSNB; incomplete type of Miyake; OMIM; 300071) (A-4: fundus photograph; A-5: OCT). One candidate disease-associated variant in the X-linked recessive gene, *CACNA1F* (OMIM; 300110), was identified in 2-IV:1.

B (Family #3): The proband (3-II:2) was diagnosed with macular degeneration with RP-like peripheral changes (B-1: fundus photograph). One homozygous disease-causing *EYS* variant was identified, together with two candidate heterozygous variants in the two autosomal dominant (AD) genes; *TOPORS* (OMIM; 609507) and *RIMS1* (OMIM; 606629). One affected daughter (3-III:1) of this proband has macular degeneration (B-2: fundus photograph). Three heterozygous variants in the *EYS*, *TOPORS* and *RIMS1* genes were identified in 3-III:1. It is still uncertain which (if any) of the two AD genes (*TOPORS* and *RIMS1*) causes the AD macular degeneration.

C (Family #4): The proband (4-III:3) was diagnosed with RP (C-1: fundus photograph). One homozygous disease-causing *EYS* variant was identified, together with one candidate homozygous variant in the autosomal recessive gene; *DRAM2* (OMIM; 613360). The clinical effects by both the *EYS* variant and the *DRAM2* variant can be considered in 4-III:3. One affected sibling (4-III:5) of this proband shows cone-rod dystrophy phenotype (C-2: fundus photograph). This subject has the heterozygous *EYS* variant and the homozygous *DRAM2* variant. The clinical effect only by the *DRAM2* variant is considered in 4-III:5.

D (Family #28): The proband (28-II:7) was diagnosed with RP (D-1: fundus photograph; D-2: FAF). Disease-causing *EYS* variants were identified in a compound heterozygous status. One sibling (28-II:6) was diagnosed with with Occult Macular Dystrophy (OMD; OMIM; 613587) (D-3: fundus photograph; D-4: FAF; D-5: OCT). One daughter (28-III:1) of this sibling (28-II:6) was also diagnosed with OMD. One disease-causing variant in the AD gene, *RP1L1* (OMIM; 608581) was identified in 28-II:6.
